# Novel splice site *IDUA* gene mutation in Tunisian pedigrees with hurler syndrome

**DOI:** 10.1186/s13000-018-0710-3

**Published:** 2018-05-29

**Authors:** Latifa Chkioua, Hela Boudabous, Ibtissem Jaballi, Oussama Grissa, Hadhami Ben Turkia, Neji Tebib, Sandrine Laradi

**Affiliations:** 1 Faculty of pharmacy, University of Monastir, 5000 Monastir, Tunisia; 2 La Rabta Hospital, 1007 Tunis, Tunisia; 3 The Auvergne-Rhône-Alpes Regional Branch of the French National Blood System EFS/GIMAP-EA-3064, 42023 Saint Etienne, France; 4 Faculty of pharmacy of Monastir, University of Monastir, Avenue Avicenne, 5019 Monastir, Tunisia

**Keywords:** Mucopolysaccharidosis type I, α-L-iduronidase, Splice site mutation, Homozygous, Compound heterozygote

## Abstract

**Background:**

The mucopolysaccharidosis type I (MPS I) is a lysosomal storage disease resulting from the defective activity of the enzyme α-L-iduronidase (*IDUA*). The disease has three major clinical subtypes (severe Hurler syndrome, intermediate Hurler–Scheie syndrome and attenuated Scheie syndrome). We aim to identify the genetic variants in MPS I patients and to investigate the effect of the novel splice site mutation on splicing of *IDUA*- *mRNA* variability using bioinformatics tools.

**Methods:**

The *IDUA* mutations were determined in four MPS I patients from four families from Northern Tunisia, by amplifying and sequencing each of the *IDUA* exons and intron–exon junctions.

**Results:**

One novel splice site *IDUA* mutation, c.1650 + 1G > T in intron 11 and two previously reported mutations, p.A75T and p.R555H, were detected. The patients in families 1 and 2 who have the Hurler phenotype were homozygotes for the novel splice site mutation c.1650 + 1G > T. The patient in family 3, who also had the Hurler phenotype, was a compound heterozygote for the novel splice site mutation c.1650 + 1G > T and for the previously reported missense mutation p.A75T. The patient in family 4 who had the Hurler–Scheie phenotype was a compound heterozygote for the novel splice site mutation c.1650 + 1G > T and for the previously reported missense mutation p.R555H. In addition, four known *IDUA* polymorphisms were identified. Bioinformatics tools allowed us to associate the variant c.1650 + 1G > T with the severe clinical phenotype of MPS I. This variant affects the essential nucleotide + 1 (G to T) of the donor splice site of *IDUA* intron 11. The G > T in intron 11 leads to wild type donor site broken with minus 19.97% value compared to normal value with 0%, hence the new splice site acceptor has plus 5.59%.

**Conclusions:**

The present findings indicate that the identified mutations facilitate the accurate carrier detection (genetic counseling of at-risk relatives) and the molecular prenatal diagnosis in Tunisia.

## Background

Mucopolysaccharidosis type I (MPS I) is an autosomal recessive lysosomal storage disorder caused by the deficient activity of the enzyme α-L-iduronidase (*IDUA*, EC 3.2.1.76). This glycosidase is required for the hydrolysis of α- L-iduronide residues of dermatan sulphate and heparan sulphate [1].

MPS I has three major clinical subtypes among which the severe Hurler syndrome (MPS IH; 607014). It is characterized by infantile onset, severe organomegaly and bone involvement, and mental retardation. The intermediate Hurler–Scheie syndrome (MPS IH/S; 607015) is characterized by onset in childhood, severe organomegaly and bone involvement, and usually limited, if any, neurological involvement. The attenuated Scheie syndrome (MPS IS; 607016) is characterized by later onset, visceral and bone disease, and neurological developmental delay [1].

The *IDUA* gene has 19 kb in length, containing 14 exons and 13 introns. It is mapped on the short arm of chromosome 4 at region p16.3 [2] being transcribed into a 2.3 kb cDNA, which encodes a 653-residue glycopeptide [3].

To date, more than 201 mutations and 32 polymorphisms have been identified [Human Gene Mutation Database] (http://www.hgmd.org; 2017). Previous mutations include 113 missense/nonsense, 33 splicing, 31 small deletions, 15 small insertions, 4 gross deletions and 3 complex rearrangements. Genetic testing MPS I patients is useful for the identification of specific genotypes, genotype-phenotype correlations and also for prenatal diagnosis.

The splice site mutations are DNA sequence changes that alter or abolish correct mRNA splicing during the process of precursor mRNA maturation. The modification in the consensus sequence, known as splice-donor and splice-acceptor sequences, which surround each exon may lead to: exon skipping, cryptic splice site activation, creation of a pseudo-exon within an intron and intron retention [4]. Hence some splice site mutations do not abolish completely the wild-type transcript expression, which may lead to less severe phenotypes [5]. In our study, we have analyzed the novel splice donor site c.1650 + 1G > T in intron 11 using bioinformatics tools to determine the impact of this variant in MPS I phenotypic expression.

## Methods

### Ethics statements

Written informed consent was obtained and signed by all studied families after a full explanation of this study, which was approved by the local ethic committees for scientific research of the La Rabta Hospital Tunis, Tunisia. Additional informed consent was obtained from all patients for whom identifying information is included in this study. All procedures were in accordance with the ethical standards of the responsible committee on human experimentation (institutional and national) and with the Helsinki Declaration.

### Study populations

This is a series of four patients (P1, P2, P3 and P4) with MPS I disease aged 2–5 years who were recruited in the pediatric department of La Rabta Hospital in Tunisia. Among the four explored MPS I families, three (family 1, family 2 and family 4) are related as second cousins. The MPS I patients had a clinical diagnosis of Hurler syndrome which was further confirmed with biological analysis by demonstrating a high excretion of GAGs in the urine and a deficiency in α-L-iduronidase activity in leukocytes. The parents and other family members of each studied family were investigated in order to create a clearer profile of the disease’s transmission to facilitate prenatal diagnosis and counseling for families at risk in Tunisia.

In this study based on clinical manifestations of MPS I patients, an analysis of urine glycosaminoglycans (GAGs) was done in first intention but this screening requires a differential diagnosis with the Hunter syndrome for which we obtained the same GAG profile.

### Biochemical diagnosis

The diagnosis of these diseases was based on the following approach after a clinical and paraclinical suspicion.

### Quantitative and qualitative analysis of total urinary glycosaminoglycans

Study of urinary glycosaminoglycans was performed first. Urinary GAGs were quantified using a dimethylmethylene blue test (DMB) [6]. The quantity of DMB bound to sulfated glycosaminoglycans was measured via spectrophotometry at wavelength of 656 nm. Electrophoresis on cellulose acetate plate was performed to identify which type of GAGs is present in excess (e.g., dermatan sulphate, heparan sulphate, keratan sulphate). Discontinuous electrophoresis on cellulose acetate plate separated the different GAGs based on their charge and differential solubility in ethanol, and the mucopolysaccharides were visualized by staining with alcian blue [6].

### Enzyme analysis

Enzyme analysis for α-L-iduronidase (MPSI, EC 3.2.1.76) was performed in sonicated leukocytes pellets as described using the 4-methylumbelliferyl-α-L-iduronide [7].

### Molecular analysis and DNA sequencing analysis

We analyzed the *IDUA* gene of 4 MPS I patients from Northern Tunisia using PCR, PCR-based restriction fragment length polymorphism (RFLP) and direct sequencing methods.

Genomic DNA was isolated from venous blood by the phenol/chloroform procedure according to standard protocols as described previously [8]. All the exons and flanking intron/exon junctions of the *IDUA* gene were amplified and sequenced. For patients with a family history of known or suspected pathogenic mutations or for the indexed cases of parents, the targeted DNA locus was analyzed.

PCR reaction consisted of 50 ng of DNA,1 X HotStarTaq buffer (Qiagen, Paris, France), 2 mM MgCl2, 200 μM of each dNTP, 10 pmol of each primer, 2.5 U of HotStarTaq (Qiagen, Paris, France) and 1 X Q solution. The final reaction volume was 18 μl. Thermal PCR profile consisted of an initial denaturation at 95 °C for 15 min, 35 cycles of denaturation at 94 °C for 30s, annealing at 68 °C for 30s and extension at 72 °C for 30s followed by a final extension step at 72 °C for 10 min. PCR products were resolved in 2% agarose gel and were visualized under UV light.

### In silico predictions

The splice site mutation was located in the defined splice site consensus sequences which were (C/A)AG|gt(a/g)agt and cag|G, for the donor splice site and the acceptor splice site, respectively [9].

We have analyzed the splice site mutation in intron 11 of *IDUA* gene by using the bioinformatics tools: Human Splicing Finder (HSF) version 2.4 [10] (http://www.umd.be/HSF/) which includes several matrices to analyze splice site and Exonic Splicing Enhancer (ESE) finder predictions (http://krainer01.cshl.edu/cgi-bin/tools/ESE3/esefinder.cgi?process=home) to examine the conservation of ESE motifs.

## Results

### Clinical features and biochemical analysis

The clinical features of each patient are presented in Table 1.

**Table 1:** Characteristics of the Tunisian MPS IH families

Family	Family 1			Family 2			Family 3			Family 4		
	Mother	Father	Patient 1	Mother	Father	Patient 2	Mother	Father	Patient 3	Mother	Father	Patient 4
Consanguinity	2nd cousins			Unrelated			1st cousins			2nd cousins		
Tunisian Origin	Nabeul			Elmaamoura			Korba			Oued Ellil		
Sex/age (Years)	36	46	M/3	32	40	F/5 (died)	37	45	M /3 (died)	34	43	M/2
Leukocyte IDUA Activity μKat/Kg Protein	Unrealized	Unrealized	0.00	Unrealized	Unrealized	0.00	Unrealized	Unrealized	0.044	Unrealized	Unrealized	0.00
Infantile onset	–	–	+	–	–	+	–	–	+	–		+
Organomegaly	–	–	Severe	–	–	Severe	–	–	Severe	–	–	Severe
Bone involvement	–	–	+	–	–	+	–	–	+	–	–	+
Mental retardation	–	–	Severe	–	–	Severe	–	–	Severe	–	–	Non marked
Growth retardation	–	–	Marked	–	–	Marked	–	–	Non marked	–	–	Non marked
Allele 1	c.1650 + 1G > T	c.1650 + 1G > T	c.1650 + 1G > T	c.1650 + 1G > T	c.1650 + 1G > T	c.1650 + 1G > T	c.1650 + 1G > T	p.A75T	c.1650 + 1G > T	c.1650 + 1G > T	p.R555H	c.1650 + 1G > T
Position	Intron 11	Intron 11	Intron 11	Intron 11	Intron 11	Intron 11	Intron 11	Exon 2	Intron 11	Intron 11	Exon 11	Intron 11
Allele 2	NL	NL	c.1650 + 1G > T	NL	NL	c.1650 + 1G > T	NL	NL	p.A75T	NL	NL	p.R555H
Position			Intron 11			Intron 11			Exon 2			Exon 11
Polymorphism	None		None			None	rs141046991		rs141046991	rs773947412	NL	rs773947412
										rs759123051	NL	rs759123051
										rs773184536	NL	rs773184536

*NL* normal, *F* female, *M* Male

**Fig. 1:**
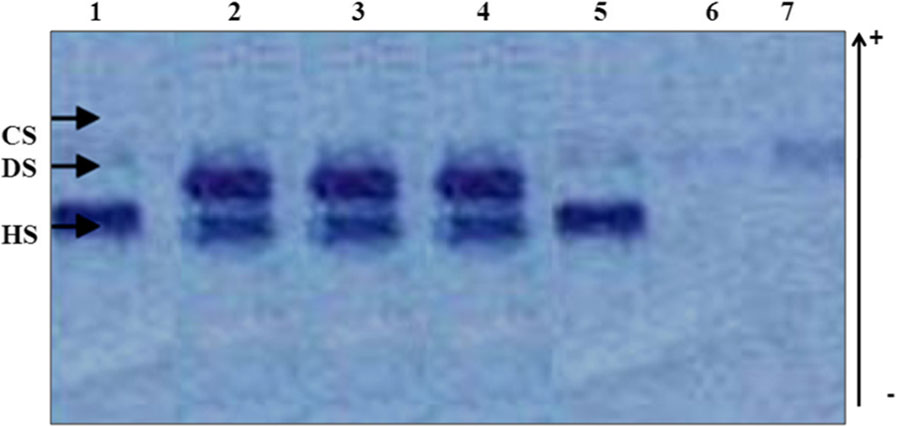
MPS I electrophoresis profile on a cellulose acetate plate of the urinary GAGs. 1 and 5: MPS III controls; 2, 3 and 4: MPS I or MPS II patients; 6 and 7: Normal controls. CS: chondroitin sulphate; DS: dermatan sulphate; HS: heparan sulphate

Phenotypic analysis confirmed the diagnosis of all the MPS IH studied patients. Indeed, for all the studied patients, the electrophoresis on cellulose acetate plate of GAGs showed the presence of heparan sulphate (HS) and dermatan sulphate (DS), an abnormal band, compared to the control case, in addition to an abnormal band of HS in MPSIII patient (Fig. 1).

*IDUA* activity in MPS IH patients ranged from 0.00 to 0.044 μKat/Kg protein.

### *IDUA* mutation analysis

Clinical and identified genotypes of studied patients are summarized in Table 1.

As a result of DNA sequencing analysis and RFLP-PCR, one novel and two previously reported mutations were identified in this study including: two missense mutations p.A75T and p.R555H, and one novel splice site mutation c.1650 + 1G > T (Table 1).

**Fig. 2:**
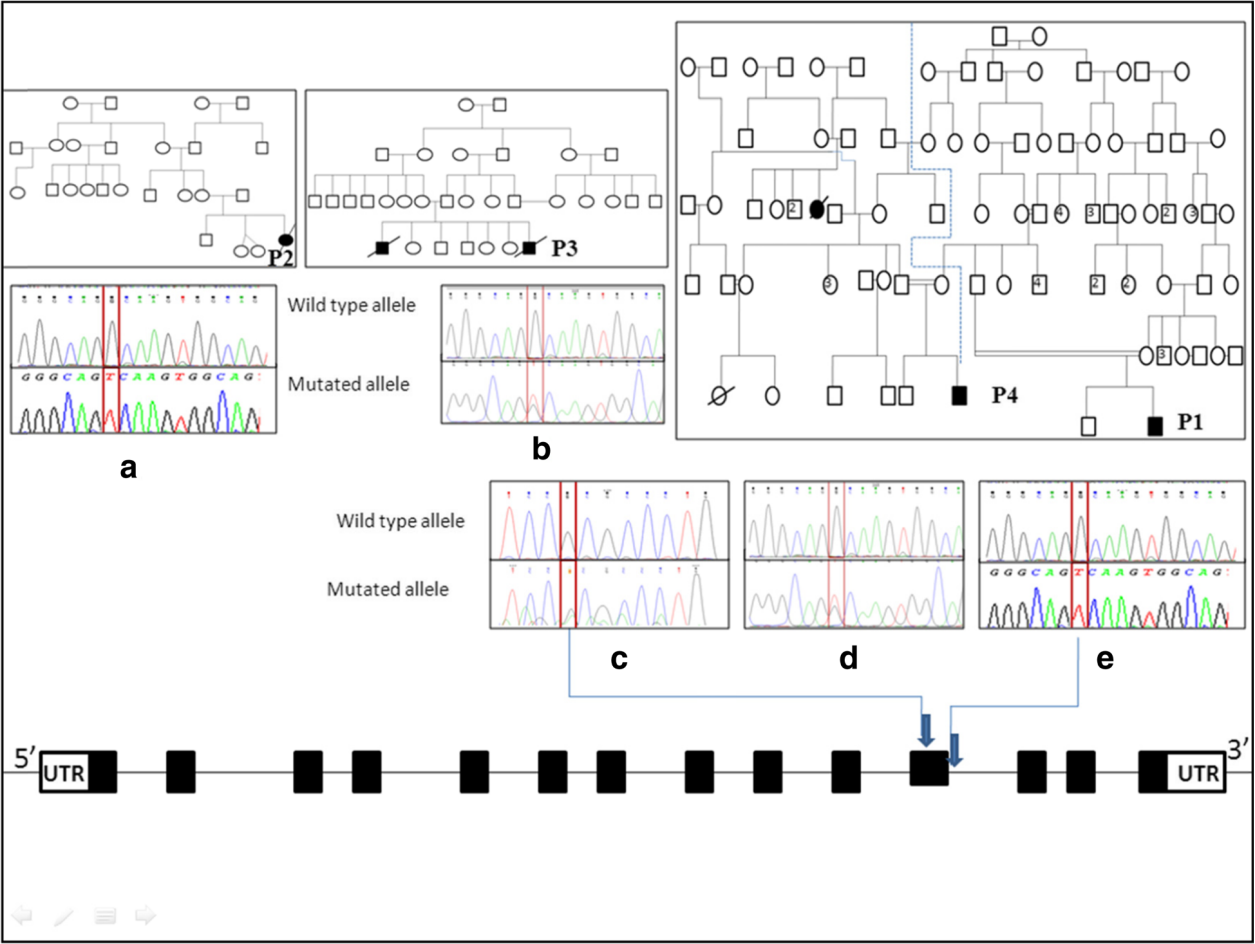
Sequence electropherograms of the *IDUA* mutations identified in the patients with Hurler syndrome: **a** and **e** showing the homozygous c.1650 + 1G > T splice site mutation (P1 and P2). **b** and **d** showing the heterozygous c.1650 + 1G > T splice site mutation (P3 and P4). **c** showing the heterozygous p.R555H missense mutation (P4)

Patients 1 and 2 from families 1, and 2 (Fig. 2) were all homozygous for a novel G to T transition in the conserved 5′ splice donor site of *IDUA* intron 11 (CAG**G**c > CAG**T**c) (Table 2). The splice site mutation obliterated a *Cac8I* restriction enzyme site. The amplicon of exon 11 from genomic DNA and its digestion with *Cac8I* resulted in three fragments (66, 101 and 378 bp) in the patients with the splice site mutation, instead of the four fragments (23, 66, 101 and 356 bp) observed in normal individuals (data not shown).

**Table 2:** Data findings: CVs and percentage variation of wild type and mutant sequences in intron 11 of *IDUA* gene (http://www.umd.be/HSF/)

Splice site type	Motif	New splice site	Wild type (WT) CV (0–100)	Mutant CV (0–100)	If cryptic site use, exon length variation (bp)	Variation (%)
Acceptor	CCGCCCGGGCAGgc	ccgcccgggcagTC	84.05	79.76	NA	−5.1
Acceptor	CGGGCAGgcaagtg	ccgggcagtcaagTG	64.94	68.57	NA	New site + 5.59
Donor	CAGgcaagt	CAGtcaagt	100	80.03	135	WT site broken −19.97
Acceptor	Ggcaagtggcagtc	gtcaagtggcagTC	73.99	77.65	NA	+ 4.95

Patient 3 was a compound heterozygote for a G to A transition in exon 2 (Fig. 2) predicting an Alanine to a Threonine missense mutation (p.A75T) and to a novel splice site mutation c.1650 + 1G > T. The p.A75T missense mutation created a *MSLI* restriction enzyme site. The *MSLI* digestion of the amplicon of exon 2 from genomic DNA resulted in two fragments (166 and 139 bp) in the patient with the missense mutation, instead of the one fragment (305 bp) observed in normal individuals (data not shown).

Patient 4 was also a compound heterozygote for a G to A transition in exon 11 (Fig. 2) predicting an Arginine to a Histidine missense mutation (p.R555H) and to the novel splice site mutation c.1650 + 1G > T.

In addition, four previously reported polymorphisms in exon 11 of *IDUA* gene were identified in the *IDUA* patients and their parents: rs141046991, rs773947412, rs759123051, rs773184536 (Table 1).

### Splice site mutation analysis

The relative strength of the splicing sites obtained from the bioinformatics tool is given as a consensual value (CVs), which varies from 0 to 100. Therefore, the effect of a splice site mutation depends on the CVs value. Splice sites with CVs over 80 are solid splicing sites, but splicing sites with CVs ranging from 65 to 70 are weak sites because only a few of these sites are active [11].

**Fig. 3:**
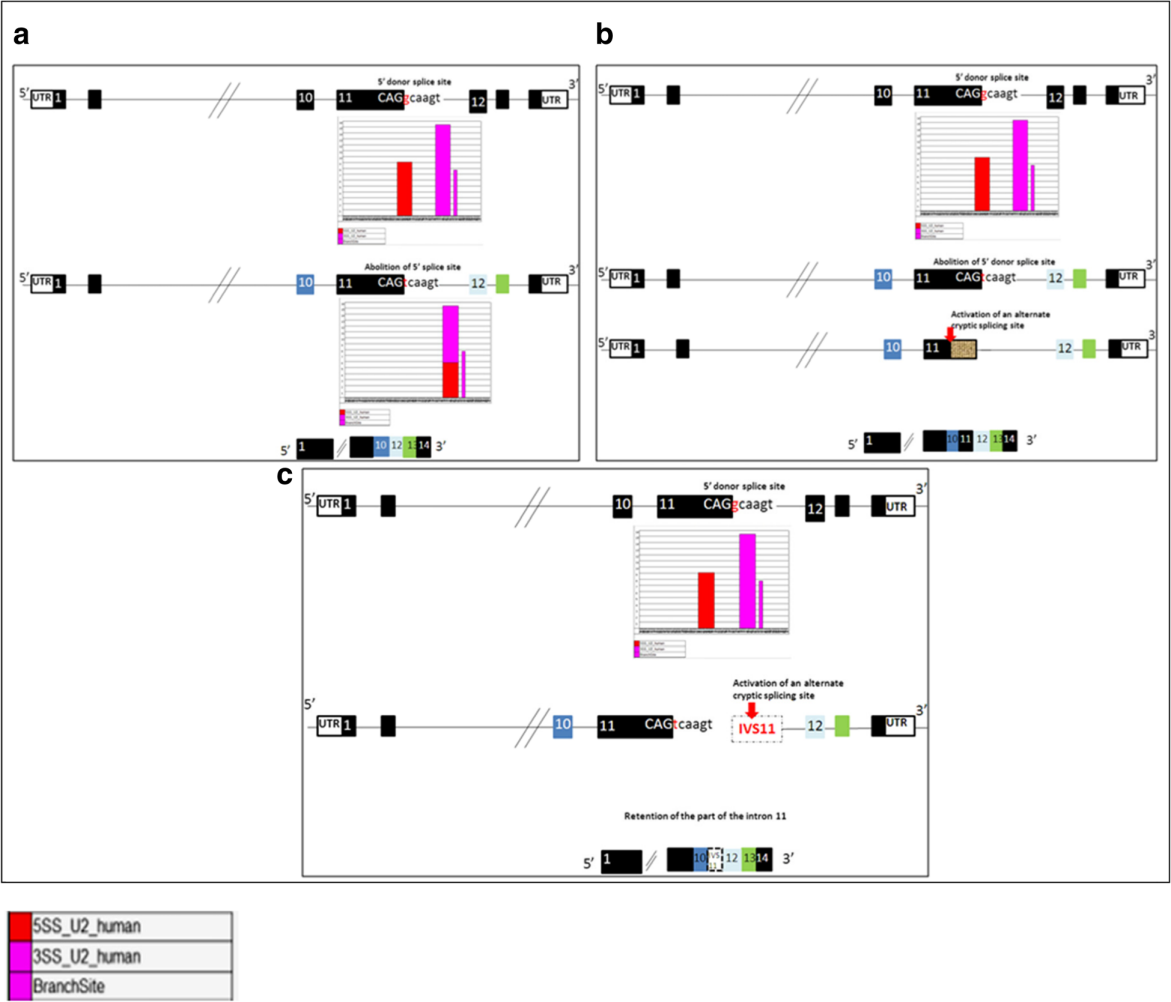
Effects of *IDUA* splice site mutation. **a** Mutation c.1650 + 1G > T, which abolies a splice donor (GT) within intron 11, resulting in skipping of the exon 11. **b** Mutation c.1650 + 1G > T, which creates an alternate cryptic splicing site, resulting in the deletion of 169 nucleotides at the end of the exon 11 giving a new size of exon 11 about 135 bp. **c** Mutation c.1650 + 1G > T which retains the part of the intron 11

The splice site mutation leads to the use of cryptic sites thus the most cryptic splice sites are to be located +/− 100 bp on each side of the exon-intron boundary. We have analyzed this region for the presence of potential splice sites using HSF version 2.4 (http://www.umd.be/HSF/) in the case of c.1650 + 1G > T mutation and we have found that the abolition of the wild type donor splice site, with minus 19.97% value, and its substitution by a new splice acceptor site, with plus 5.59% value, leads to three possible conclusions (Fig. 3): firstly, the loss of exon 11; secondly, the retention of the part of the intron 11 sequence; and finally, the activation of an alternative cryptic splicing site in exon 11 giving a new size of exon 11 of about 135 bp compared to the normal length of 304 bp (Table 2).

The results using the Human Splicing Finder, showed that the wild phenotype has a donor splice site CVs close to 100, thus this new splice site could be strong and functional enough to justify the mutant phenotype which presented a new splice acceptor site with a CV at about 80 which would justify the new isoform of mRNA in patients (Table 2).

## Discussion

This work was conducted as a straight continuation of studies carried out in other Tunisian MPS I patients and their families [12–15]. In this cohort we were interested in patients presenting a severe phenotype of MPS I. All MPS IH patients have the splice site c.1650 + 1G > T mutant allele in homozygous and/or heterozygous forms.

Our study showed that all studied patients were from different regions within the Northern part of Tunisia: Tunis (Oued Ellil) and Nabeul (Korba and Elmaamoura) that are over 60 km away from each other; however, three families (P1, P2 and P4) were known to be related.

Patients 1 and 2 (P1 and P2) who developed a severe form of MPS I were homozygotes for the novel splice site mutation c.1650 + 1G > T.

Our results about P1 and P2 using in silico predictions showed that the wild donor splice site and the mutant splice site have higher CVs and below 80 respectively.

A splice site mutation may cause activation of an alternative cryptic splice site, preferred to the use of the legitimate splice site. The G > T mutation at the 5′-donor splice site of intron 11 presumably causes exon skipping, the loss of exon 11, and subsequently an aberrant polypeptide that is misfolded (http://rulai.cshl.edu/tools/ESE3) which should explain the severe phenotype of MPS I. In further studies, we may suggest to perform in vitro analysis in order to confirm the in silico predictions and therefore i) to study the impact of the splice site mutation during the process of precursor mRNA maturation, ii) to analyze the phenotypic expression of this disease.

Prenatal diagnosis was performed in family 1. The fetus was found homozygous for the splice site mutation however the parents refused the interruption of pregnancy. Then, the patient 1 was included in the register of patients wishing to perform a bone marrow transplant but neither matching relative nor an unrelated matched donor has been found so far.

The P3 of family 3 does not present any relationship with the other investigated families but he lives in the same region of Tunisia. He was found to be a compound heterozygote for the novel splice site mutation and the previously reported missense mutation p.A75T. He developed severe features at an early age (9 months); this finding is in agreement with those reported in the literature [16]. The severe phenotype observed in this patient, results from the association of both mutated alleles: c.1650 + 1G > T and p.A75T. Thus, this new splice site appears to be strong and functional enough to justify the mutant phenotype observed in P3 which presents a new splice acceptor site with a CV at about 80 and which would justify the new isoform of mRNA in this patient. The p.A75T mutation has also an impact on phenotype manifestation. This genetic lesion was a non conservative mutation resulting from a change of a non polar Alanine to a polar Threonine at position 75 of *IDUA* protein. The p.A75T mutation was described for the first time in a patient with a severe MPS I in North America [17]. Patient 3 died at the age of three and his brother, who developed the similar severe clinical phenotype died before our molecular investigation; he probably had the same genetic lesion as patient P3.

Family 4 appears to present a relationship with the other studied families (1 and 2), as second cousins. P4 developed early clinical manifestations at 9 months of age, e.g. dysmorphic facial appearance and hepatosplenomegaly, hence these features were presented in moderate form at this age. Patient 4 was a compound heterozygote for the novel splice site mutation and the previously reported missense mutation p.R555H. The molecular analysis of genomic DNA of parents confirmed the segregation of the mutants’ alleles. His parents were heterozygous for the splice site mutation c.1650 + 1G > T and for the missense mutation p.R555H, respectively.

The p.R555H missense mutation in exon 11 occurred at a CpG dinucleotide, a hotspot for mutation [18], and resulted in a nonconservative transition of a basic Arginine to a neutral Histidine. Homozygous patients for the p.R555H mutation have been reported from European countries in patients with the severe phenotype.

The investigation of Tunisian patients suffering from Hurler disease allowed us to define a geographical distribution of the *IDUA* mutations. Thus, the missense mutation p.P533R was recurrent in MPS I patients of Southern Tunisia [11] whereas the splice site mutation c.1650 + 1G > T seems to be frequent in Northern Tunisia. Neither of these mutations have been identified in a different territory from those in their regions of origin. It is noteworthy that in the Maghreb (Morocco, Tunisia) only the p.P533R-MPS I missense mutation has been identified so far [10, 11].

Unfortunately, some MPS I patients die before their investigations. This can be explained by the clinical difficulty and thus the delay of diagnosis of the MPS I inherited disease. The adverse socioeconomic conditions of those patients make the situation even more difficult.

## Conclusion

The novel mutation expands the *IDUA* gene mutation spectrum and contributes to the recognition of its impact on phenotypic expression in MPS I patients. The geographical distribution of these most frequent mutations observed in MPS I patients- the novel splice site mutation c.1650 + 1G > T and the missense mutation p.P533R in Northern and Southern of Tunisia respectively- will be useful for the identification of the carrier.

## Abbreviations

DMB: Dimethylmethylene blue test
GAGs: Glycosaminoglycans
HSF: Human Splicing Finder
*IDUA*: α-L-iduronidase
MPS IH: Mucopolysaccharidosis type IH
MPSI: Mucopolysaccharidosis type I
WT: Wild type
